# A systematic analysis of the skeletal muscle miRNA transcriptome of chicken varieties with divergent skeletal muscle growth identifies novel miRNAs and differentially expressed miRNAs

**DOI:** 10.1186/1471-2164-12-186

**Published:** 2011-04-13

**Authors:** Tingting Li, Rimao Wu, Yong Zhang, Dahai Zhu

**Affiliations:** 1 National Laboratory of Medical Molecular Biology, Institute of Basic Medical Sciences, Chinese Academy of Medical Sciences, School of Basic Medicine, Peking Union Medical College, Beijing, China; 2 Department of Biomedical Informatics, Peking University Health Science Center, 38 Xueyuan Rd, Beijing 100191, China; 3 Institute of Systems Biomedicine, Peking University Health Science Center, 38 Xueyuan Rd, Beijing 100191, China

## Abstract

**Background:**

Functional studies have demonstrated that microRNAs (miRNAs or miRs) play critical roles in a wide spectrum of biological processes including development and disease pathogenesis. To investigate the functional roles that miRNAs play during chicken skeletal muscle development, the miRNA transcriptomes of skeletal muscles from broiler and layer chickens were profiled using Solexa deep sequencing.

**Results:**

Some miRNAs have multiple isoforms and several miRNAs* are present at higher levels than their corresponding miRNAs. Thirty three novel and 189 known chicken miRNAs were identified using computational approaches. Subsequent miRNA transcriptome comparisons and real-time PCR validation experiments revealed 17 miRNAs that were differentially expressed between broilers and layers, and a number of targets of these miRNAs have been implicated in myogenesis regulation. Using integrative miRNA target-prediction and network-analysis approaches an interaction network of differentially expressed and muscle-related miRNAs and their putative targets was constructed, and miRNAs that could contribute to the divergent muscle growth of broiler and layer chickens by targeting the *ACVR2B *gene were identified, which can causes dramatic increases in muscle mass.

**Conclusions:**

The present study provides the first transcriptome profiling-based evaluation of miRNA function during skeletal muscle development in chicken. Systematic predictions aided the identification of potential miRNAs and their targets, which could contribute to divergent muscle growth in broiler and layer chickens. Furthermore, these predictions generated information that can be utilized in further research investigating the involvement of interaction networks, containing miRNAs and their targets, in the regulation of muscle development.

## Background

Embryonic patterning and organogenesis involve coordinated differentiation, migration, proliferation and programmed cell death in metazoans. These complex cellular and developmental processes rely on precise spatiotemporal networks that regulate transcription factors at multiple levels including mRNA transcription and translation, protein stability and degradation. Recently, evidence has demonstrated that microRNAs (miRNAs or miRs) are involved in diverse aspects of biology including developmental regulation and the pathogenesis of human diseases [1-4]. miRNAs are small 19-24 nucleotide (nt) regulatory RNAs that generally modulate gene expression through translational repression or by causing deadenylation and degradation of target mRNAs [5,6]. However, miRNAs could function as activators to regulate gene expression [7,8]. The biogenesis of miRNAs is spatiotemporally regulated by various mechanisms [9], providing additional evidence that miRNAs are functionally significant, and potentially key regulators of gene expression during development [10-15].

An essential role for miRNAs in terms of regulating skeletal muscle development is evident from studies demonstrating that deletion of a conditional *Dicer *allele in embryonic skeletal muscle results in perinatal lethality due to skeletal muscle hypoplasia [16]. In particular, the critical roles of three muscle-specific miRNAs, miR-1, miR-133 and miR-206, in the regulation of myogenesis have been well documented [17,18]. miR-1 and miR-133 have been reported to regulate different aspects of skeletal muscle development *in vitro *and *in vivo *[19]. miR-1 promotes myocyte differentiation by repressing the expression of histone deacetylase 4 (HDAC4), a negative regulator of differentiation and a repressor of the MEF2 (myocyte enhancer factor-2) transcription factor [19]. In C_2_C_12 _myoblasts, miR-133a promotes proliferation, in part, by repressing serum response factor (SRF) [19]. Like miR-1, miR-206 promotes differentiation of C_2_C_12 _myoblasts *in vitro*. miR-206 induces muscle differentiation by repressing the expression of the DNA polymerase α subunit (Pola1) [20], connexin 43 (Cx43) [21], follistatin-like 1 (Fstl1) and utrophin (Utrn) [22]. In addition to muscle-specific miRNAs, several ubiquitously expressed miRNAs have a role to play during muscle development. For example, zebrafish miR-214 was reported to regulate the slow muscle phenotype by targeting *suppressor of fused *(*Sufu*), a negative regulator of hedgehog signaling [23]. Expression of miR-181 isoforms, miR-181a and miR-181b, are induced upon initiation of myogenesis and they participate in the regulation of myoblast differentiation by repressing HoxA-11 protein levels [24]. The functional significance of miRNAs in terms of controlling myogenesis has been documented, but the majority of miRNAs are abundantly expressed. Therefore, identifying novel miRNAs that are expressed at low levels during skeletal muscle development but are functionally important requires robust approaches such as high-throughput deep sequencing technology.

The chicken (*Gallus gallus*) is an established model organism for studying vertebrate development, primarily because chicken embryos are readily accessible and easily manipulated [25]. In addition, a variety of standard chicken breeds with different phenotypes are readily available, which collectively represent a valuable genetic resource. Broiler chickens (bred for meat production) and layer chickens (bred for egg production) are ideal model systems for studying the molecular mechanisms underlying myogenesis [26]. During the past 80 years, genetic selection in broilers has concentrated on a high growth rate and large muscle mass; in contrast, layers have been selected for egg production. Therefore, even under optimal growth conditions, the body size of layers is smaller than that of broilers owing to intrinsic genetic differences between the two varieties. These unique biological features of broilers and layers allow muscle development to be investigated. In previous studies, we have successfully identified protein-coding and non-coding genes with roles during myogenesis using broilers and layers as model systems [27,28].

In the current study, previous work was expanded to identify miRNAs involved in myogenesis regulation by comparing the miRNAs transcriptome in skeletal muscle tissues of broilers and layers. Solexa deep sequencing was carried out to profile miRNAs expressed in chicken skeletal muscle tissues. Sequence-tag analyses have shown that a group of highly abundant, known miRNAs are expressed in skeletal muscles and 33 novel putative chicken miRNAs from skeletal muscle tissues have been identified. Comparing the expression patterns of known and novel miRNAs demonstrated that they were significantly differentially expressed between broiler and layer chicken muscle tissues. These results were confirmed using microarrays and real-time reverse transcription-polymerase chain reaction (RT-PCR) validation experiments. Of the 17 miRNAs examined using RT-PCR, nine presented with an expression pattern consistent with the microarray analysis; 15 miRNAs had a pattern consistent with the deep sequencing data. Using computational prediction, targets for these differentially expressed miRNAs and muscle-related miRNAs were identified, and an interaction network was constructed. Furthermore, miR-1 was demonstrated specifically to target the 3' untranslated region of the activin receptor IIB gene, *ACVR2B*, which can cause dramatic increases in muscle mass [29]. This integrative analysis highlights the complexity of gene expression networks regulated by microRNAs in muscle cells during muscle development.

## Results

### Characterization of the miRNA transcriptome of skeletal muscle from broiler and layer chickens using deep sequencing

**Figure 1:**
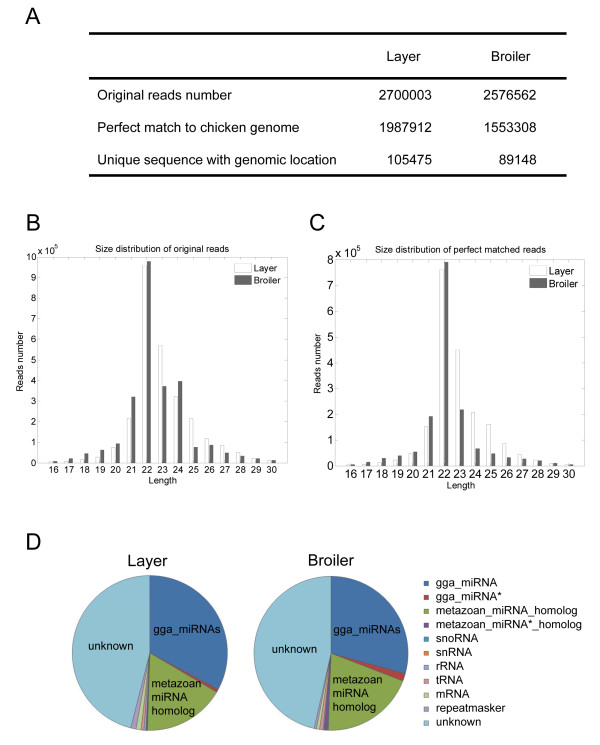
**Deep sequencing results and annotations of small RNAs from chicken skeletal muscle**. ***A. ***Number of small RNA reads from broilers and layers. ***B, C. ***Size distribution of sequenced small RNAs. ***D. ***Annotations of sequenced small RNAs.

Solexa sequencing was used to profile miRNAs expressed in layer and broiler chicken skeletal muscles. Sequencing of a small RNA fraction (16-30 nt) from total RNA extracted from pectoralis muscles collected from 10-day-old chicken embryos yielded 2,700,003 and 2,576,562 reads for the layer and broiler libraries, respectively (Figure 1A). Of these, 1,987,912 layer sequences and 1,553,308 broiler sequences, which account for more than 67% of the total reads, were perfectly mapped to the chicken genome (May 2006). The sequencing data were simplified by grouping all identical sequence reads together; therefore, 105,475 unique layer sequences and 89,148 unique broiler sequences were used for subsequent analysis (Figure 1A).

The most abundant size class in the small RNA sequences distribution was 22 nt, followed by 21 and 23 nt (Figure 1B and 1C), and this was consistent with the known 21-23 nt range for miRNAs. To assess the efficiency of deep sequencing for miRNA detection, all sequence reads were annotated and classified by analyzing the sequence tags in relation to the data from miRBase (version 16), RefSeq mRNA, RepeatMasker and non-protein-coding RNAs annotated by ENSEMBL. The sequence tag annotations demonstrated that known chicken miRNAs (gga_miRNAs) and metazoan miRNA homologs accounted for ~50% of all sequence reads in the broiler and layer libraries (Figure 1D). These results indicate that the deep sequencing data were highly enriched for mature miRNA sequences, suggesting that the data are reliable for expression profiling of known miRNAs and deep mining for novel miRNAs.

**Figure 2:**
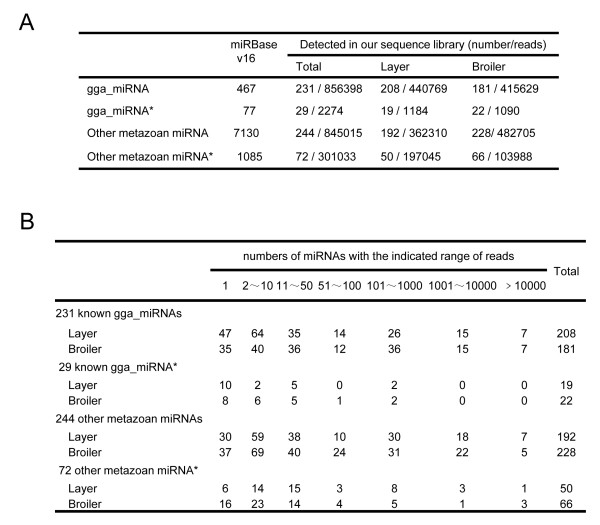
**Known miRNAs and homologs of metazoan miRNAs detected in chicken skeletal muscle**. ***A. ***Numbers of known miRNAs and chicken homologs of metazoan miRNAs detected by perfect match in the present study. ***B. ***Reads distribution of known miRNAs and chicken homologs of metazoan miRNAs in chicken skeletal muscle.

To investigate the expression of known miRNAs in broiler and layer skeletal muscles, the numbers and distribution of small RNA sequences that matched known chicken miRNA genes were analyzed. The results demonstrated that of 467 known chicken miRNAs and 77 miRNA*s in the miRBase (version 16), perfect matches to 231 miRNAs and 29 miRNA*s were obtained in the sequencing data (Figure 2A and Additional file 1). Among the sequences that were not perfectly matched to known chicken miRNAs or miRNA*s there were 244 metazoan miRNA homologs and 72 metazoan miRNA* homologs (Figure 2A and Additional file 2).

As presented in Additional file 5 and 6, known miRNAs and metazoan homologs had a broad range of expression levels in skeletal muscle tissues, ranging from hundreds of thousands of sequence reads for the most abundant miRNAs to single reads for the least abundant. The distribution of read numbers for the known miRNAs is summarized in Figure 2B. The 33 most abundant miRNAs (i.e. those with > 1,000 reads) are presented in Table 1.

**Table 1:** The most abundant miRNAs in chicken skeletal muscles as determined using deep sequencing

miRNA ID	Layer	Broiler	B/L	Studies related to skeletal muscle
gga-miR-206	222998	131609	0.59	[20-22]
gga-let-7c	32663	87111	2.67	
gga-miR-103	59224	37050	0.63	[19]
gga-let-7j	26141	42513	1.63	
gga-let-7f	24694	16121	0.65	
gga-miR-221	18238	5340	0.29	[33,84]
gga-miR-107	10815	10353	0.96	[85]
gga-miR-130a	2630	10933	4.16	[19]
gga-let-7b	4832	8155	1.69	
gga-miR-128	5059	6669	1.32	[15]
gga-miR-130b	1409	9002	6.39	[19]
gga-miR-222	3735	4013	1.07	[33,84]
gga-miR-16c	1209	6058	5.01	[39]
gga-miR-15b	875	5759	6.58	
gga-miR-125b	1506	4801	3.19	[40]
gga-miR-21	1056	5128	4.86	[19,39,86]
gga-miR-101	3647	1305	0.36	
gga-miR-181a	2015	2543	1.26	[24,85]
gga-miR-130c	795	2872	3.61	[19]
gga-miR-99a	1589	1561	0.98	[19,87]
gga-miR-1a	979	1495	1.53	[19]
gga-miR-456	1601	592	0.37	
gga-miR-148a	1180	738	0.63	
gga-miR-30a-3p	1189	422	0.35	[19]
gga-miR-146c	1067	309	0.29	[88,89]
gga-miR-460	307	914	2.98	
gga-miR-181b	421	795	1.89	[24]
gga-miR-10a	890	269	0.30	[41]
gga-miR-20b	210	940	4.48	[19]
gga-miR-15c	91	1038	11.4	
gga-let-7i	479	632	1.32	
gga-let-7k	506	602	1.19	
gga-miR-383	886	219	0.25	

### miRNA transcriptome analysis demonstrated the presence of several highly abundant miRNAs in skeletal muscles of broilers and layers

Almost all known muscle-specific miRNAs (myomiRs) were represented among the more abundant miRNAs identified. The most abundant miRNA was gga-miR-206, which was represented by approximately 200,000 sequence reads in the broiler and layer libraries (Table 1). The predominance of miR-206 is consistent with its well established function during skeletal muscle development [30] and reported role during chicken myogenesis [31,32]. Two other myomiRs, miR-1 [19] and miR-181 [24], were high-count sequences in both libraries (Table 1). Compared with the three myomiRs, forms of the myomiR miR-133 were expressed at low levels in the skeletal muscle libraries: there were 126 reads for gga-miR-133a in the layers library and 67 in the broilers library; 7 reads for gga-miR-133b in the layers library and 12 in the broilers library; one gga-miR-133c read in the layers library and no read in the broilers library (Additional file 1). These variations in abundance could reflect differences in the roles of these miRs in terms of the regulation of myogenesis [19,24]. In addition to miR-206, miR-1 and miR-181, nine other miRNAs among the most abundant in these libraries (miR-221, miR-222, miR-21, miR-103, miR-130, miR-99, miR-30, miR20, and miR128) have been implicated in the proliferation and differentiation of muscle cells (Table 1) [15,19,33]. Therefore, the miRNA transcriptome for layers and broilers revealed by this analysis is highly enriched for miRNAs involved in myogenesis regulation.

### The expression patterns of several miRNA*s during chicken skeletal muscle development are unique

A total of 29 miRNA*s were detected in broiler and layer libraries (Figure 2A and 2B). The majority were expressed at low levels (Additional file 1), but of those expressed at higher levels, such as miR-181a and miR-1677, the read counts were significantly lower than those of the corresponding miRNAs (Table 2). One striking exception to this general trend was gga-miR-140*; this was present as 911 reads in the layers and 711 reads in the broilers libraries, but gga-miR-140 was not detected in either library (Table 2), suggesting that gga-miR-140* functions during chicken skeletal muscle development. gga-miR-126* is another case in which only miRNA* was detected (Table 2). There were several cases, such as miR-199 and miR-1329, where miRNA and miRNA* were generated at similar levels (Table 2).

**Table 2:** A comparison of read counts between miRNA and the corresponding miRNA*

miRNA ID	miRNA		miRNA*	
		
	Layer	Broiler	Layer	Broiler
gga-miR-181a	2015	2543	142	32
gga-miR-1677	429	184	20	76
gga-miR-199	91	318	26	133
gga-miR-1329	16	123	36	44
gga-miR-140	0	0	911	711
gga-miR-126	0	0	11	28

### Analysis of sequence variants indicated that many miRNAs possess isomiRs

**Figure 3:**
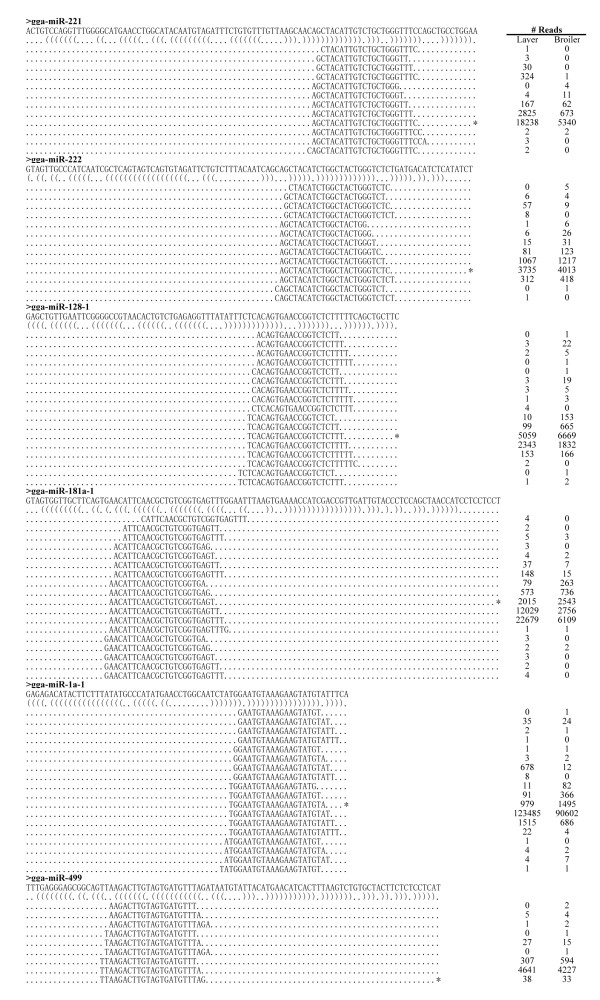
**IsomiRs from several gga-miRs**. Reads alignments of the various isoforms of several gga-miRs are presented. The sequence of the gga-miR hairpin is presented in the top line; the brackets below denote the secondary structure. Reads that aligned with the mature gga-miR sequence as reported in miRBase are denoted by a series of asterisks. The number of reads corresponding to each sequence is presented on the right.

As found in previous deep sequencing studies, heterogeneity at the 5' and/or 3' ends of miRNAs was observed (Figure 3, Additional file 3). miRNAs with such variations from their miRBase reference sequences are referred to as isomiRs [34,35]; some typical examples are presented in Figure 3. In the majority of cases (e.g. gga-miR-221) the most abundant isoform is identical to the reference in miRBase (Figure 3). In some cases, such as gga-miR-222 and gga-miR-128, more than one highly abundant isoform was present (Figure 3), indicating that some miRNAs have more than one functional isoform in specific tissues/organs. For some miRNAs, such as gga-miR-181a, gga-miR-1a and gga-miR-499, the most abundant isoform was not among the known miRNA sequences reported in miRBase 16 (Figure 3). A similar phenomenon has previously been observed for miRNAs identified in chicken embryos [36], suggesting that a refinement to the miRBase annotations for chicken miRNAs is required to reflect experimentally observed abundances.

### Novel miRNAs are less abundant and less evolutionarily conserved in chicken skeletal muscle

In addition to profiling known miRNAs, deep sequencing is a powerful strategy for discovering novel miRNAs that may not have been detected using traditional methods for sequencing cDNA libraries. Using the miRDeep program as a predictive tool [37], 33 putative novel chicken miRNAs were obtained from broiler and layer sequence tags (Table 3, Additional file 4). Genomic sequence analyses demonstrated that six of these putative miRNAs were located in the exons of annotated genes, fourteen resided in the introns of annotated genes and thirteen were present in intergenic regions (Additional file 5). The putative novel miRNAs were less abundant (Table 3) than known miRNAs (Figure 2A and 2B). Only one novel putative miRNA had read counts greater than 100 in the library (Table 3).

**Table 3:** Novel chicken miRNAs predicted by miRDeep

miRNA ID	chr	strand	start	end	Layer*	Broiler*
gga-miR-N1	chr3	+	4348337	4348358	135	62
gga-miR-N2	chrUn_random	-	13277374	13277395	2	52
gga-miR-N3	chrUn_random	+	30704197	30704218	29	21
gga-miR-N4	chr17	+	2477065	2477087	18	14
gga-miR-N5	chr27	+	1117232	1117255	22	6
gga-miR-N6	chr2	+	103042957	103042979	26	0
gga-miR-N7	chr18	-	5042929	5042952	20	5
gga-miR-N8	chr4	-	3214463	3214484	23	0
gga-miR-N9	chr6	+	31550357	31550378	18	4
gga-miR-N10	chr1	-	180215042	180215065	14	3
gga-miR-N11	chr4	-	51257851	51257872	7	9
gga-miR-N12	chrZ	-	28247215	28247238	12	4
gga-miR-N13	chr3	+	85117942	85117965	13	1
gga-miR-N14	chr4	+	563352	563373	11	3
gga-miR-N15	chr26	-	2669812	2669834	11	2
gga-miR-N16	chr3	+	59307968	59307990	5	7
gga-miR-N17	chr18	-	4116255	4116279	6	5
gga-miR-N18	chr3	+	724977	724994	0	7
gga-miR-N19	chr4	+	2151238	2151261	4	3
gga-miR-N20	chr7	-	37893831	37893851	6	1
gga-miR-N21	chr19	+	4862173	4862194	1	4
gga-miR-N22	chr20	-	10896392	10896413	5	0
gga-miR-N23	chr15	-	11255901	11255922	4	0
gga-miR-N24	chr24	+	2618818	2618840	3	1
gga-miR-N25	chr27	-	4426392	4426414	4	0
gga-miR-N26	chr4	+	16625715	16625736	3	1
gga-miR-N27	chrUn_random	-	40250702	40250728	3	1
gga-miR-N28	chr1	+	52701699	52701723	2	1
gga-miR-N29	chr10	-	16415151	16415173	1	1
gga-miR-N30	chr2	+	133303756	133303776	1	1
gga-miR-N31	chr27	-	3957524	3957545	2	0
gga-miR-N32	chr3	-	49536173	49536194	1	1
gga-miR-N33	chr7	+	12850964	12850988	0	2

*Sequenced read-numbers are presented.

To investigate evolutionary conservation of the 33 novel chicken miRNAs, a search for highly similar sequences among human, mouse, rat, opossum, frog and zebrafish genomic sequences was carried out using a BLAST analysis. Obtaining mature miRNAs from homology sequences does not necessarily signify that the miRNAs are conserved as they might not be capable of forming hairpin structures. We further identified hairpin-like RNA structures using RNAfold (see Materials and Methods). The same analysis was carried out for known chicken miRNAs. The results demonstrated that novel miRNAs are less evolutionarily conserved (Additional file 6), a result that is consistent with previous studies [36]. Further analyses using a multiple alignment of six vertebrate genomes in the UCSC database with that of the chicken genome revealed that only gga-miR-N3 was conserved in at least one of the analyzed vertebrate genomes. gga-miR-N3 exists in zebrafish and frogs but is lost after the emergence of mammalian lineages (Additional file 7). The remaining 32 miRNAs could be avian- and/or chicken-specific miRNAs. To identify potential chicken-specific miRNAs, the sequences were checked against the Zebra Finch (*Taeniopygia guttata*) Alignment Net Track in UCSC. Nineteen of the 32 novel chicken miRNAs were present in the Zebra Finch, suggesting that the remaining 13 novel miRNAs could be specific to the chicken lineage (Additional file 8). Combining conservation and relative abundance information for the newly identified and known miRNAs revealed that the evolutionarily conserved miRNAs were among the most abundant, supporting a correlation between evolutionary conservation and the expression level of miRNAs.

### Identification of differentially expressed miRNAs in broiler and layer skeletal muscle

**Figure 4:**
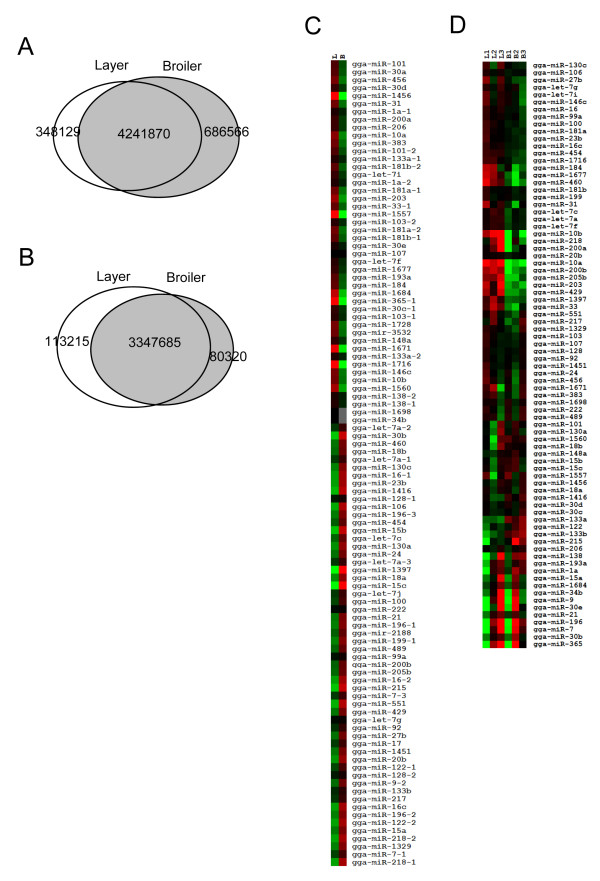
**miRNAs differentially expressed in the skeletal muscles of broilers and layers**. ***A. ***Venn diagram demonstrating the overlap of original sequenced reads between broilers and layers. ***B. ***Venn diagram demonstrating the overlap of sequenced reads with perfect genomic matches in broilers and layers. ***C. ***Heat-map of miRNAs differentially expressed in broilers and layers based on the read counts obtained by deep sequencing. ***D. ***Heat-map of differentially expressed miRNAs confirmed by microarrays. Triplicate samples of total RNA from the skeletal muscle of E10 broilers and layers were used to perform miRNA microarray experiments. B, broiler chicken; L, layer chicken.

The main objective of the present study was to identify miRNAs involved during skeletal muscle development by comparing skeletal muscle miRNA transcriptomes in broilers and layers. Analysis of sequencing results demonstrated that more than 80% of reads overlapped between broilers and layers (Figure 4A). The overlap between libraries was greater (94%) for those reads with perfect genomic matches (Figure 4B), suggesting that the deep sequencing data were reliable for direct comparison of miRNA abundance between broilers and layers.

In addition to the 33 novel miRNAs, 189 known miRNAs were identified in miRBase using miRDeep (Additional file 9). Comparing Table S6 with Table S1 demonstrates that the number of reads could differ for the same miRNA. In Table S1, the reads number for each miRNA is based on perfect matches to known chicken miRNAs in miRBase. As presented in Figure 3 and Additional file 3, many miRNAs have different isomiRs in addition to perfect matches. Counting only perfect-match isoforms may not be appropriate as the isoform listed in the miRBase may not be the only functional isoform. In miRDeep, different isoforms of the same miRNA are counted together. To arrive at the figure of 189 known miRNAs identified by miRDeep, the DEGseq package [38] was used to identify differentially expressed miRNAs on the basis of potentially significant changes in relative miRNA abundance between broilers and layers. Expression of 102 miRNAs was significantly different between broilers and layers (Additional file 10, Figure 4C). miRNA microarrays were employed to characterize the expression profiles of these 102 differentially expressed miRNAs further (Additional file 10, Figure 4D).

**Figure 5:**
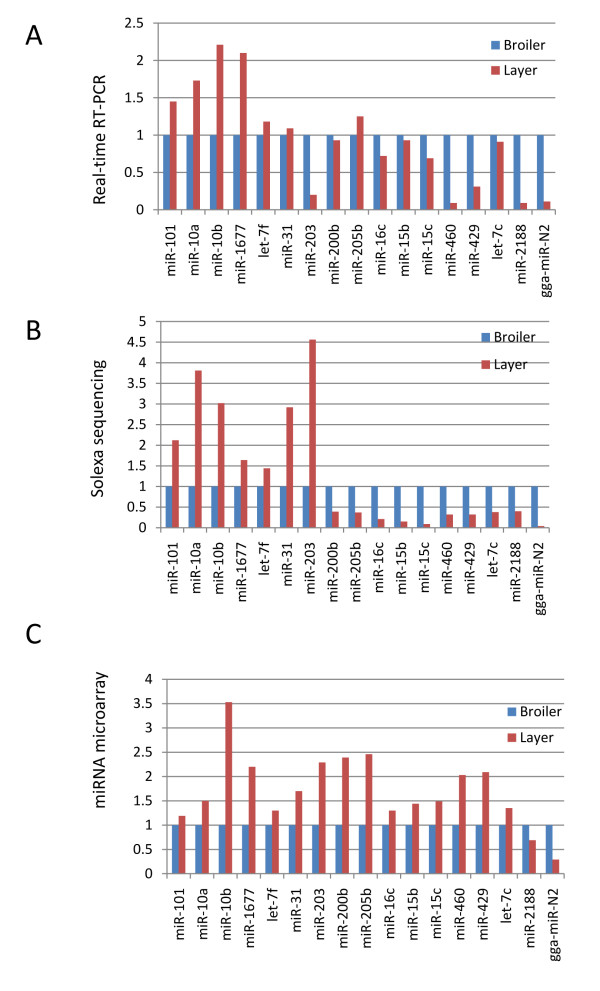
**Validation of differentially expressed miRNAs using real-time RT-PCR**. ***A. ***Real-time RT-PCR results for 17 miRNAs that were differentially expressed in broilers and layers. ***B. ***Relative abundance of 17 miRNAs in skeletal muscles of broilers and layers based on deep sequencing data. ***C. ***Expression pattern of 17 miRNAs in skeletal muscles of broilers and layers based on microarray experiments.

To validate the differential expression of these miRNAs between broilers and layers, 17 miRNAs were randomly selected and their expression levels quantified using real-time RT-PCR (Figure 5). Of the 17 miRNAs examined, nine (52.9%) had an expression pattern consistent with the microarray analysis (Figure 5A and 5C), and 15 miRNAs (88.2%) presented with a pattern consistent with the deep sequencing data (Figure 5A and 5B). These data provide evidence that deep sequencing is a more sensitive and reliable method for identifying differentially expressed miRNAs than miRNA microarrays.

### Target prediction and network analysis highlight the complexity of interactions among miRNAs and their targets during muscle development

**Figure 6:**
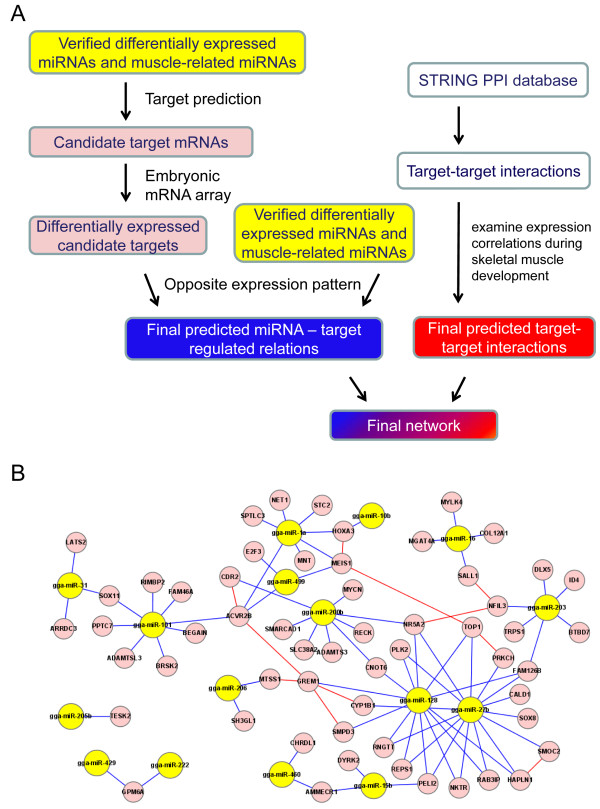
**Interaction network of differentially expressed miRNAs and their candidate targets**. ***A***. Workflow of interaction network analysis. Network construction can be divided into two components: miRNA-target interactions and target-target interactions. For miRNA-target interactions, candidate miRNA targets were predicted by TargetScan. Differentially expressed candidate targets were identified using mRNA microarrays that covered embryonic days 10, 12, 14 and 18. The final miRNA-target relations correspond to those mRNAs differentially expressed between broiler and layer that exhibited a pattern of expression opposite to that of the corresponding miRNAs. Target-target interaction pairs were extracted from the STRING database. A pairwise PCC was then calculated for each pair based on transcription profiles during skeletal muscle development to extract putative target-target interactions. ***B***. The final integrated network. In the network, miRNAs are represented by yellow nodes and targets are represented by pink nodes. Blue lines denote miRNA-target interactions and red lines denote target-target interactions.

Of the 17 differentially expressed miRNAs confirmed using real-time RT-PCR, six have been functionally linked to myogenesis [39-42]. However, the majority including one novel miRNAs (gga-miR-N2) and eight known miRNAs (miR-101, miR-15b, miR-15c, miR-1677, miR-200, miR-460, gga-mir-2188 and miR-429) have not been implicated in the regulation of muscle development. To approach the question of how miRNAs could function in concert with their target genes in terms of controlling muscle development and to provide some molecular insight into the process, targets of the miRNAs were identified and a possible regulatory network of interactions among miRNAs and their targets was constructed. The strategy and workflow are summarized in Figure 6A.

The starting point of the miRNA target prediction strategy was the 16 validated, differentially expressed, known miRNAs and eight other muscle-related miRNAs. TargetScan (version 5.1) [43] was used to predict the putative targets for these 24 miRNAs, identifying more than 1000 annotated mRNA transcripts that were potential targets (Additional file 11). The mechanism of miRNA function predicts that miRNAs and their targets normally exhibit correlated expression patterns [44]. Therefore, an mRNA transcriptome analysis was performed using microarrays to identify mRNAs in embryonic skeletal muscle that were differentially expressed between broilers and layers. In addition to analyzing mRNA transcriptomes on embryonic day 10 (E10), as was done for the miRNA transcriptome, embryos were analyzed on embryonic days 12, 14 and 18 (E12, E14 and E18), yielding a total of 1057 non-redundant genes that were differentially expressed between broilers and layers (Additional file 12). To narrow the field of candidate targets for the 24 miRNAs further, the analysis was restricted to those miRNA targets that were differentially expressed between broilers and layers and had an expression pattern opposite to that of the corresponding miRNAs. Using these criteria, 57 candidate targets for 16 miRNAs were identified (Additional file 13) and used for subsequent network analysis.

In addition to interacting with miRNAs, targets should interact with each other. To investigate such interactions, protein-protein interactions (PPIs) of these putative targets were extracted from the STRING database. Information concerning protein-protein interactions in bird species in the current PPIs database is very limited. Therefore, PPI data for human orthologs of these miRNA targets were utilized. It is widely accepted that some human PPIs may not be conserved in chickens, and protein interactions are time- and condition-specific. To establish more reliable interactions, we applied a previously proposed referencing strategy in which PPIs are filtered with dynamic gene expression patterns [45,46]. The chicken gene expression dataset contained 54 microarrays that covered nine developmental stages during skeletal muscle development of broiler and layer chickens (see Materials and Methods). To identify putative target-target interactions, the Pearson Correlation Coefficient (PCC), which is known to provide information about the "shape" of gene expression changes [47,48], was used; an absolute PCC value of 0.3 was used as a cutoff.

miRNA-target interactions and target-target interactions were integrated to construct possible regulatory networks (Figure 6B). One of the predicted miRNA-target relationships presented in Figure 6B, between miR-27b and the target *CYP1B1*, has been reported previously [49]. The remaining relationships are reported for the first time; therefore, this analysis predicts several candidates for future studies concerning miRNA-target function in controlling muscle development. In the presented network, one major regulatory module involved 13 miRNAs (yellow nodes) and 55 targets (pink nodes). Of these 13 miRNAs, five (miR-206, miR-1a, miR499, miR-128 and miR-27b) have been reported to have a role during muscle development [30,50]. Little is known about the functional roles of the remaining eight (miR-31, miR-101, miR-200b, miR-10b, miR-460, miR-15b, miR-16 and miR-203) during muscle development. However, analysis of their targets demonstrated that several were involved in myogenesis regulation, suggesting that these miRNAs could participate in regulating muscle development through their target genes. For example, miR-200b has eight predicted targets, among which are three genes *RECK, SLC38A2 *and *Nr5a2*, which encode proteins that are reported to be involved in muscle development [51-53].

**Figure 7:**
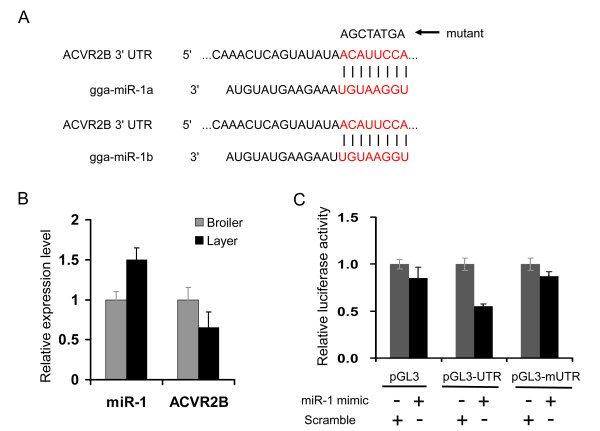
**miR-1 directly targets the chicken *ACVR2B *UTR**. ***A. ***Schema of miR-1 binding site in chicken *ACVR2B *3'-UTR sequence (seed sequence highlighted in red). Mutated *ACVR2B *3'-UTR eliminates the seed binding site pointed by arrow. ***B. ***Expression of miR-1 and *ACVR2B *gene in skeletal muscle of broiler and layer chickens at embryonic day 18 were analyzed using real-time RT-PCR. ***C. ***Target validation using a luciferase reporter assay. 293T cells were co-transfected with miR-1 mimic or scramble double-strand small RNA and the reporter plasmid pGL3 or pGL3-*ACVR2B*-UTR or pGL3-m*ACVR2B*-UTR, respectively.

It has been reported that activin A receptor type IIB (ACVR2B) plays an important role in regulating muscle development by interacting with a number of transforming growth factor-β (TGF-β) family members [54,55]. ACVR2B causes dramatic increases in muscle mass (up to 60% in two weeks) when injected into wild-type mice [29]. No miRNAs have been identified previously as regulatory factors for *ACVR2B*, but the network analysis predicted that *ACVR2B *is a target of three miRNAs: gga-miR-101, gga-miR-1a and gga-miR-499 (Figure 6B). It has been demonstrated that miR-1 is an important regulator of myogenesis [19,56]. miR-1 and *ACVR2B *had opposite expression patterns in skeletal muscle tissue from broiler and layer chickens (Figure 7B). Therefore, the target relationship between miR-1 and *ACVR2B *was validated using a luciferase reporter gene assay. As demonstrated in Figure 7C, the luciferase activity was significantly reduced when a miR-1 mimic was co-transfected with pGL3-*ACVR2B*-UTR containing a miR-1 targeting site into 293T cells, suggesting that miR-1 directly targets chicken *ACVR2B *UTR. Therefore, it is conceivable that miRNAs could be involved in regulating ACVR2B function in terms of controlling muscle development. Taken together, the results of the network analysis suggest that myogenesis is regulated by a complicated network, mediated by multiple miRNAs acting through the same target gene, and/or single miRNAs targeting multiple genes.

## Discussion

Recent developments in high-throughput sequencing technology have enabled the miRNA transcriptome to be profiled in various organisms [36,57,58]. In the present study, Solexa deep sequencing was used to provide an extensive miRNA profile of the previously unexamined skeletal muscle of broiler and layer chicken lines. The sequence analysis identified 33 novel chicken miRNAs and demonstrated that many miRNA precursors could generate multiple isoforms (isomiRs). Importantly, a comparison of miRNA transcriptomes allowed us to identify 16 known miRNAs and one novel miRNA that were differentially expressed in the skeletal muscles of broilers and layers. On the basis of the predicted targets of the 16 differentially expressed known miRNAs and eight muscle-related miRNAs, an interaction network comprising these miRNAs and their candidate targets was constructed.

The data presented in this report provide the first miRNA transcriptome profile of chicken skeletal muscle. Two hundred and thirty one known miRNAs and 29 miRNA*s were detected in skeletal muscles. gga-miR-206 was the most abundant miRNA in skeletal muscles of broilers (131,609 reads) and layers (222,998 reads), a result that is consistent with the well-established function of miR-206 during skeletal muscle development [30]. Interestingly, Rathjen and colleagues recently performed miRNA profiling in chicken somites and demonstrated that among the 85 detectable known miRNAs, gga-miR-10b was the most abundant (113,106 reads), whereas gga-miR-206 was much less abundant (259 reads) [59]. Taken together, these observations suggest that miR-206 and miR-10b could play important roles at different stages during muscle development. The expression level of myomiR miR-133 was lower than miR-206 in the skeletal muscle library, an outcome that could reflect differences in the roles of these miRNAs in terms of myogenesis regulation. Consistent with this interpretation, miR-206 has been shown to promote skeletal muscle differentiation, whereas miR-133 regulates myogenesis by increasing muscle cell proliferation [19].

In addition to well-known myomiRs, recent studies have demonstrated that several other miRNAs are involved in regulating myogenesis. For example, a comparison of miRNA expression profiles in proliferating myoblasts and differentiated myotubes revealed that miR-221 and miR-222 are down-regulated upon differentiation of primary and established myogenic cells, whereas miR-21, miR-103, miR-130, miR-99, miR-30 and miR20 are up-regulated [19,33], suggesting that these miRNAs play important roles in the transition between proliferation and differentiation of muscle cells. Interestingly, these same eight miRNAs were abundantly expressed in our sequencing libraries, indicating that they could play regulatory roles in controlling the difference in skeletal muscle growth rates between broilers and layers during development.

Seventeen miRNAs that were differentially expressed in the skeletal muscle of broiler and layer chickens were identified. Seven (miR-101, miR-10a, miR-10b, miR-1677, let-7f, miR-31, and miR-205b) were expressed at higher levels in layers, and ten (miR-203, miR-200b, miR-16c, miR-15b, miR-15c, miR-460, miR-429, let-7c, miR-2188, and gga-miR-N2) were expressed at higher levels in broilers. Six of these miRNAs (miR-31, miR-10a, miR-10b, miR-16C and two let-7 members) have been implicated in skeletal muscle regeneration or development [39-42]. Greco and colleagues demonstrated that miR-31 was induced in dystrophic (mdx) mice and in Duchenne muscular dystrophy patients, and in newborn mice and newly formed myofibers during postischemic regeneration, suggesting that it could be important in pathophysiological pathways that regulate muscle responses to damage and regeneration [42]. Recent studies have reported that miR-10 contributes to retinoic acid-induced smooth muscle cell differentiation [41], and may be important during the early stage of embryonic myogenesis [59]. Taken together, the approaches used in this study have identified a number of differentially expressed miRNAs that could exert novel functions in terms of regulating muscle cell proliferation and differentiation during development. Further investigations concerning the function of these miRNAs should facilitate our understanding of the regulatory roles of miRNAs in terms of controlling the divergent skeletal muscle growth rates of broiler and layer chickens.

miRNAs exert their effects by interacting with target mRNAs. Therefore, target-predicting software (TargetScan) was used to identify putative targets of these differentially expressed miRNAs and eight muscle-related miRNAs, before an interaction network of these miRNAs and their candidate targets was constructed. The interaction networks predicted that *ACVR2B *is a target of gga-miR-101, gga-miR-1a and gga-miR-499. Prior to this analysis, there have been no reports concerning associations between *ACVR2B *and miRNAs. The ACVR2B receptor signaling pathway mediates the function of myostatin [60] and can regulate muscle growth *in vivo *[29]. *ACVR2B *haplotypes have been reported to be associated with muscle mass and strength in humans [60]. Furthermore, acute inhibition of myostatin/ACVR2B signaling with the antagonist ACVR2B-Fc preserves skeletal muscle in mouse models of cancer cachexia [61]. *ACVR2B *was expressed at higher levels in the skeletal muscle of broilers than in layers at E18, indicating that *ACVR2B *could be related to the higher growth rate of broiler skeletal muscle. The results of this analysis indicate that the three putative miRNA regulators of *ACVR2B *may be involved in this process. The analysis demonstrated that ACVR2B interacts with two other targets, CDR2 and GREM1. *GERM1*, a putative target of gga-miR-128, encodes a protein that is a BMP4 antagonist and an effective regulator of myogenic progenitor proliferation [62].

*RECK*, the putative target of gga-miR-200b, is down-regulated by MyoD to facilitate myotube formation, and up-regulated by MRF4 to promote other aspects of myogenesis [51]. *SLC38A2*, encoding a sodium-coupled amino acid transporter, is the putative target of gga-miR-200b. SLC38A2 regulates proteolysis through phosphoinositol 3-kinase, and provides a link among acidosis, insulin resistance and protein wasting in skeletal muscle cells [52]. *SOX8*, the putative target of gga-miR-27b, acts as a specific negative regulator of skeletal muscle differentiation, possibly by interfering with the function of myogenic basic helix-loop-helix proteins [63]. *MEIS1 *is the putative target gene of gga-miR-1a and gga-miR-499. MEIS1, together with PBX1A, facilitates binding of MyoD (a family of transcription factors with the remarkable ability to induce myogenesis *in vitro *and *in vivo*) to non-canonical E boxes in the myogenin gene to induce myogenesis [64]. Furthermore, a putative MEIS1 binding site is located in the minimal promoter of myostatin [65]. The *CALD1 *gene, the putative target of gga-miR-27b, encodes two caldesmon-1 isoforms through alternative splicing: high molecular mass CaD (h-CaD), which is exclusively expressed in smooth muscle, and low molecular mass CaD (l-CaD), which is ubiquitously expressed in all cell types except skeletal muscle. The h-CaD/l-CaD ratio can be used as a marker to monitor differentiating and pathological states of smooth muscles [66]. *LATS2*, a putative target of gga-miR-31, encodes a protein that has been reported to regulate the size of myocytes in the heart negatively [67]. *Nr5a2 *is the putative target of gga-miR-200b, gga-miR-128 and gga-miR-27b. Its product, the nuclear receptor transcription factor Nr5a2, has been reported to function during skeletal muscle organization [53]. Therefore, although little is known about the specific functions of several of these miRNAs (e.g. miR-31, miR-101, miR-200b, miR-10b, miR-460, miR-15b, miR-16 and miR203) during muscle development, the close relationship between their targets and myogenesis regulation demonstrates a potential role during muscle development.

Given the significant functions of miRNAs in various biological processes, it is perhaps not surprising that miRNA biogenesis is tightly regulated at each stage of miRNA generation; in particular, a number of studies have highlighted the complexity of post-transcriptional processing [9]. Sequence variations in mature miRNAs attributable to heterogeneity at the 5' and 3'-ends creates an additional level of complexity in miRNA processing [36]. A majority of miRNA genes have strand bias [68]. In some cases, miRNA genes have been found to generate similar amounts of miRNAs and their corresponding miRNA*s [36]. The analysis of deep sequence tags identified several miRNAs that had read counts similar to those of their corresponding miRNA*s, suggesting that these genes encode miRNAs on both arms of the precursor in skeletal muscle tissues. Although the functional significance of this observation has not been established experimentally, the fact that the miRNA and its miRNA* are co-expressed at similar levels indicates that miRNAs serve independent functions in cells.

This study identified 33 novel chicken miRNAs and analysis of the evolutionary conservation of these newly identified miRNAs revealed that only one is conserved in non-avian vertebrates and the remaining 32 are likely to be specific to bird and/or chicken lineages. Few newly identified miRNAs are conserved among vertebrates, whereas the majority of known miRNAs identified using traditional cloning methods are abundantly expressed and relatively conserved during evolution [69-71]. Further support for this observation was provided by a recent report concerning the identification of miRNAs in various organisms using high-throughput sequencing. This approach demonstrated that most newly identified miRNAs discovered using deep sequencing are present only in a small group of organisms [36]. Therefore, it is reasonable to hypothesize that these non-conserved miRNAs could play important roles in establishing and maintaining phenotypic diversity among different groups of organisms during evolution. In addition, the bird and/or chicken lineage miRNAs reported in this study could have arisen during genetic selection, and function as key regulators of the differences in growth rates between broiler and layer chickens. A functional examination of novel and species-specific miRNAs is a challenge for future research and an important step in improving our understanding of the critical roles played by miRNAs during development and evolution.

## Conclusions

The present study is the first to examine the chicken skeletal muscle miRNA transcriptome, and to evaluate miRNA function during skeletal muscle development through the identification of differentially expressed miRNAs between broiler and layer chickens, which have divergent skeletal muscle growth. Identification of novel miRNAs highlights the important function of low abundance and less conserved miRNAs during development of specific tissues. To investigate the functional roles of miRNAs during chicken skeletal muscle development, an interaction network of the differentially expressed miRNAs and their putative targets was constructed. This integrated analysis provides information that will aid further experimental investigations concerning miRNAs and their targets during skeletal muscle development.

## Materials and methods

### Chicken embryo incubation and tissue collection

Meat-type broiler eggs (Arbor Acres) and egg-type layer eggs (White Leghorn) were incubated at 37.5°C for 10 or 18 days (E10 or E18). For Solexa sequencing and miRNA microarray analysis, skeletal muscles (*pectoralis*) were collected from broilers and layers at E10; for mRNA microarray analysis, muscles were collected at E10, E12, E14 and E18. Muscle samples were immediately frozen in liquid nitrogen and stored at -80°C pending RNA isolation. For analysis of the tissue expression pattern of novel miRNAs, tissues (brain, heart, liver, lung, breast muscle, intestine, kidney, fat, and stomach) were collected from layer chickens at E18. All embryonic manipulations were conducted in accordance with the protocols of the Chinese Academy of Medical Sciences and the Institutional Animal Care and Use Committee of Peking Union Medical College.

### Small RNA library construction and sequencing

Total RNA was isolated from skeletal muscles using TRIzol reagent (Invitrogen) and precipitated overnight at -20°C. Approximately 20 μg of total RNA from broiler and layer chickens was submitted to the Beijing Genomics Institute (BGI) for Solexa sequencing. In brief, sequencing was performed by fractionating total RNA using polyacrylamide gel electrophoresis (PAGE) to enrich for molecules in the range of 16-30 nt, and then ligated with proprietary adapters. Following adaptor ligation, cDNA was synthesized from total RNA by reverse transcription and amplified with 15 PCR cycles to produce libraries for sequencing.

### Analysis of sequencing data

After filtering low-quality reads and trimming the adaptor sequences, totals of 2,700,003 and 2,576,562 reads were obtained for layers and broilers, respectively. Sequencing data were simplified by grouping identical sequence reads together, yielding 827,431 unique sequences. The unique sequence reads were mapped to the UCSC chicken genome galGal3 by ZOOM; 168,642 uniq reads, corresponding to 3,541,220 sequences, were mapped to the genome with an exact match.

The various types of ncRNAs or degradation products in the sequence library (Figure 1D) were annotated by reference to miRNAs from miRBase (version 16); coding exons based on RefSeq mRNA and repeat sequences based on RepeatMasker were obtained from UCSC; snoRNA, snRNA, rRNA and tRNA were based on Ensembl ncRNA data (version 54). Metazoan miRNA homologs of chicken miRNAs were identified in *Anopheles gambiae, Ateles geoffroyi, Apis mellifera, Bombyx mori, Bos taurus, Caenorhabditis briggsae, Caenorhabditis elegans, Canis familiaris, Drosophila melanogaster, Danio rerio, Fugu rubripes, Gorilla gorilla, Homo sapiens, Lemur catta, Lagothrix lagotricha, Monodelphis domestica, Macaca mulatta, Mus musculus, Macaca nemestrina, Ovis aries, Pan paniscus, Pongo pygmaeus, Pan troglodytes, Rattus norvegicus, Saguinus labiatus, Sus scrofa, Tetraodon nigroviridis, Xenopus laevis *and *Xenopus tropicalis*. Sequencing reads representing miRNA sequences often have untemplated nucleotides in the 3' end [72,73]. Therefore, miRNAs were annotated by identifying tags that were exactly matched to the 5' 19 nt of known miRNAs. An analysis of the size distribution of these 5' end-matched sequences indicated that the most abundant size was 22 nt (Additional file 14). Those 20-25 nt tags whose 5' 19 nt matched the 5' 19 nt of known miRNAs were counted as copies of known miRNAs. The numbers of chicken miRNA*s, and miRNAs and miRNA*s of other metazoans, were established using the same criterion. Other ncRNAs including snoRNA, snRNA, rRNA and tRNA, repeat sequences and mRNA degradation products were annotated on the basis of perfect matches. The annotation order was chicken miRNA, chicken miRNA*, metazoan miRNA homolog, metazoan miRNA* homolog, snoRNA, snRNA, rRNA, tRNA, mRNA, and RepeatMasker. After each annotation step, only unmatched reads were used for the next annotation step.

Simple perfect matches to miRBase (version 16) sequences were used to determine the 467 known chicken miRNAs and 77 miRNA*s and their expression patterns (read numbers) in the data sets (Figure 2A and Additional file 5). For sequences that were not perfectly matched to known chicken miRNAs or miRNA*s, metazoan miRNA homologs and metazoan miRNA* homologs were identified using perfect sequence matches (Figure 2A and Additional file 6).

The deep sequencing data obtained were deposited in the GEO database with the accession number GSE20942.

### Prediction of novel miRNAs

Novel miRNAs were identified using the miRDeep package described by Friedlander et al., which can effectively distinguish miRNAs from other kinds of ncRNAs [37]. All sequences were mapped to the chicken genome (gal3) using megaBLAST, and only exactly matched sequences were retained for further analysis. As some miRNAs could lie in exonic regions of mRNAs [74,75], reads that aligned to more than five genomic positions or RepeatMasker annotation files were discarded. The remaining aligned reads were used as a reference, and potential precursor sequences were extracted from the chicken genome. The secondary structures of potential precursor sequences were predicted by RNAfold [76]. A FASTA file containing known mature metazoan miRNA sequences in miRBase was used as input to allow for conservation scoring. Using miRDeep, 222 putative miRNAs were obtained, 189 of which mapped to known miRNAs in the chicken genome. The remaining 33 were novel chicken miRNAs (Table 3). Using the same cutoff on a permuted dataset, 16 putative miRNAs were obtained. Therefore, the corresponding signal-to-noise ratio was approximately 14:1 (222/16).

### Conservation analysis of miRNAs

Genomic sequences for six vertebrates (hg18, mm8, rn4, monDom4, xenTro2 and danRer4) were downloaded from the UCSC genome browser. BLASTN was used to identify regions of homology to chicken miRNA sequences in these genomes. From the BLAST results, sequences that covered more than 80% of the queried mature miRNA sequences and had fewer than two mismatches in the covered region were selected. The seed regions of the miRNAs are more conserved, therefore covered regions were required to start from the 5' end. Finding "hit" sequences to mature miRNAs does not necessarily signify that the miRNAs are conserved as they may not be capable of forming hairpin structures. Accordingly, candidate sequences were extracted and all hairpin-like RNA structures encompassing small RNA sequence tags identified using RNAfold. Hairpin-like RNAs, whose mature miRNA regions were more than 70% matched with the miRNA* regions, were accepted as homologs of chicken miRNAs. The conservation heat-map was constructed using Cluster 3.0 [77] and visualized in TreeView 1.60. In the heat-map (Additional file 2), the dark color represents 0, signifying that no homologs were identified in the corresponding species. The red color represents 1, which indicates the presence of homologs in the corresponding species.

### Identification of differentially expressed miRNAs on the basis of deep sequencing data

miRNAs expressed at significantly different levels in broilers and layers were identified using the DEGseq package [38]. Using the likelihood ratio test model, proposed by Marioni et al. [78], and a cutoff of 1 × 10^-4^, 102 known miRNAs were identified as being significantly differentially expressed.

### miRNA microarray

Custom-designed miRNA microarrays were synthesized in situ by LC Sciences (Houston, USA) and used to analyze miRNA expression patterns in the skeletal muscle of broilers and layers. Arrays contained 1721 DNA probes including a non-redundant set of probes complementary to 440 known chicken miRNAs and 78 to known chicken miRNA*s. Arrays included probes for 78 predicted chicken snoRNAs, positive control probes for chicken U6 snRNA, 1124 unknown chicken small RNAs and negative controls for normalizing data with low-density signals. Hybridizations and scans were performed by LC Sciences; microarrays were scanned using an Axon GenePix 4000B Microarray Scanner. Data among arrays were normalized using a cyclic LOWESS (locally weighted regression) method [79]. The microarray data obtained were deposited in the GEO database with the accession number GSE20947.

### Quantitative real time RT-PCR

Differentially expressed miRNAs identified using deep sequencing and microarrays were validated by stem-loop RT-PCR [80] using the stem-loop RT-PCR primers presented in Additional file 15. Total RNA was isolated from skeletal muscles using TRIzol (Invitrogen), and genomic DNA contamination was removed by digesting with DNase I at 37°C for 30-40 min. DNase-treated RNAs were extracted using phenol/chloroform and precipitated with ethanol. Reverse transcriptase reactions contained RNA samples, 50 nM stem-loop RT primer, 1 × RT buffer, 0.25 mM each dNTPs, 3.33 U/ml MultiScribe reverse transcriptase and 0.25 U/ml RNase inhibitor. Reaction mixtures were incubated in a 9700 Thermocycler for 30 min at 16°C, 30 min at 42°C, 5 min at 85°C, and held at 4°C. Reverse transcriptase reactions including no-template controls and RT-minus controls were run in duplicate. Real-time PCR was performed using a standard SYBR Green PCR Master Mix (ABI). The reaction mixtures were incubated in a 96-well plate at 95°C for 10 min, followed by 40 cycles of 95°C for 15 s and 60°C for 1 min. All reactions were run in triplicate.

### Network construction

Network construction was divided into two components: miRNA-target interactions and target-target interactions. Starting with 16 known miRNAs validated as differentially expressed between broilers and layers, and eight muscle-related miRNAs (gga-miR-1, gga-miR-206, gga-miR-499, gga-miR-221, gga-miR-222, gga-miR-128, gga-miR-367 and gga-miR-27b), TargetScan (version 5.1) [43] was used to predict putative targets. Given the reported inverse correlation between miRNA and target expression patterns [44], the analysis was restricted to those differentially expressed miRNA targets whose mRNA expression pattern opposed that of the corresponding miRNAs. Expression levels of mRNA targets of miRNAs were measured using commercial Affymetrix Chicken Genome Arrays. Microarray experiments were carried out by CapitalBio Corporation (Beijing, China). Total RNA from skeletal muscles collected from broilers and layers at E10, E12, E14 and E18, and prepared as described above, was hybridized in triplicate with three biological repeats from broilers and layers at each developmental stage. Therefore, a total of 24 microarrays were used in the present study. Normalization was performed using RMA [81] software with a CDF file annotated by Dai et al. [82]. The Affymetrix GeneChip is a commonly used microarray platform for genome-wide expression studies. However, several genes/transcripts on the arrays are out of date owing to updates in genome assemblies, causing problems when mapping the probes to new versions of the genome assembly [83]. To solve this problem, Dai et al. [82] aligned the probes to different sources of genome data to filter out problematic probes. The original. CEL files were re-annotated in the present study using annotations generated by Dai et al., ultimately obtaining 12,495 probe sets corresponding to 12,495 Entrez genes. Entrez genes whose expression values changed more than 1.5 fold between broilers and layers were selected for each of four time points. To filter out genes that did not have identical expression profiles in each group, t-tests (p-value < 0.05) were used to obtain a final differentially expressed gene list. The microarray data obtained were deposited in the GEO database with the accession number GSE20990.

In addition to interactions between miRNAs and their targets, interactions between miRNA targets were identified. To establish target-target interactions, PPI data from the STRING database (version 8.0) were downloaded. The chicken PPI data were limited, so human ortholog PPI data were used for these miRNA targets, applying a previously proposed referencing strategy that filtered the PPIs with gene expression patterns [45,46] to increase the reliability of the predicted interactions. The gene expression dataset used here contained nine time points: E10, E12, E14, E18, Day1 (day of birth), W2 (postnatal week 2), W4, W6 and W8 for broilers and layers. For each time point, there were three biological repeats for a total of 54 microarrays (Additional file 16). The generation of E10, E12, E14 and E18 expression data is described above. Day 1, W2, W4, W6 and W8 expression data were generated in a previous study [27]. For each target-target interaction pair extracted from the STRING database, a PCC value was calculated on the basis of the expression profiles from E10 to W8. An absolute PCC value of 0.3 (p-value < 0.05) was used as a cutoff to establish putative target-target interactions.

### Target validation using a luciferase reporter gene assay

A pGL3-control vector (pGL3) was used for 3' UTR-luciferase reporter assays. The TargetScan Human database http://www.targetscan.org/ was used to identify the predicted miR-1 binding site. 3' UTR fragment of chicken *ACVR2B *containing a miR-1 binding site was amplified from chicken genomic DNA with primers [F: GCTCTAGAGCTGGCCAGTTTTGAAGCAGAGGC (Xba I) and R: GCTCTAGAGCCCCCTGCTCACGGCTGTTGG (Xba I)] and cloned downstream of the luciferase gene to create the pGL3-luc-*ACVR2B*-UTR constructs. miR-1 seed region mutations were generated by site-directed mutagenesis with primers (mut-F: CAAACTCAGTATATAAGCTATGAGTAAGGTTAGTATTGCAAAAC and mut-R: GCAATACTAACCTTACTCATAGCTTATATACTGAGTTTGATTGGT). Reporter assays were conducted in triplicate using 293T cells in 24-well plates. Transfections were performed with 150 ng of reporter plasmid and 50 ng of miR-1 mimic or scramble (Fugene; Roche). A pRL-TK reporter was used as an internal control to normalize for transfection efficiencies.

## Authors' contributions

TL carried out the bioinformatics analysis and drafted the manuscript. RW carried out animal care and tissue sampling and performed the experiments. YZ carried out the experimental design and drafted the manuscript. DZ conceived the study and participated in its design and coordination, and helped to draft the manuscript. All authors read and approved the final manuscript.

## Supplementary Material

Additional file 1 Table S1: Reads number and sequences of the 231 chicken miRNAs and 29 chicken miRNA*s which were obtained in the sequencing data by perfect matches. Click here for file

Additional file 2 **Table S2**: Reads number and sequences of the 244 metazoan miRNA homologs and 72 metazoan miRNA* homologs which were obtained in the sequencing data by perfect matches. Click here for file

Additional file 3 **Table S3: IsomiRs for some known miRNAs**. Click here for file

Additional file 4 **Figure S1: Secondary structures of 33 putative novel chicken miRNAs**. Click here for file

Additional file 5 **Table S4:Genomic locations of the novel miRNAs**. Click here for file

Additional file 6 **Figure S2:The conservation heat-maps for the novel and known chicken miRNAs**. Click here for file

Additional file 7 **Figure S3: UCSC genome browser tracks showing the conservation of gga-miR-N3**. Click here for file

Additional file 8 **Table S5: Reads number and sequences of the avian-specific and chicken-specific novel miRNAs**. Click here for file

Additional file 9 **Table S6:Reads number of 189 known miRNAs got by miRDeep**. Click here for file

Additional file 10 **Table S7: Reads number and expression levels of the 102 differentially expressed miRNAs got by deep sequencing**. Click here for file

Additional file 11 **Table S8: The predicted targets for 16 differentially expressed miRNAs and eight muscle-related miRNAs by TargetScan**. Click here for file

Additional file 12 **Table S9: Differentially expressed genes between broilers and layers on embryonic day 10, 12, 14, and 18, respectively**. Click here for file

Additional file 13 **Table S10: miRNA-target and target-target interactions in the network**. Click here for file

Additional file 14 **Figure S4: Size distribution of reads whose 5' 19 nt were exactly matched to the 5' 19 nt of known miRNAs**. Click here for file

Additional file 15 **Table S11:Primers for miRNA detection by real-time RT-PCR**. Click here for file

Additional file 16 Table S12: **The gene expression dataset for broilers and layers at nine time points: E10, E12, E14, E18, Day1 (day of birth), W2 (postnatal week 2), W4, W6 and W8. For each time point, there were three biological repeats**. Click here for file
