# Supplementary material for: A systematic analysis of the skeletal muscle miRNA transcriptome of chicken varieties with divergent skeletal muscle growth identifies novel miRNAs and differentially expressed miRNAs
Source: BMC Genomics. 2011 Apr 13;12:186. doi: 10.1186/1471-2164-12-186 (PMC3107184; doi:10.1186/1471-2164-12-186)
Supplement: Additional file 6 — Figure S2:The conservation heat-maps for the novel and known chicken miRNAs. [file 1471-2164-12-186-S6.DOC]

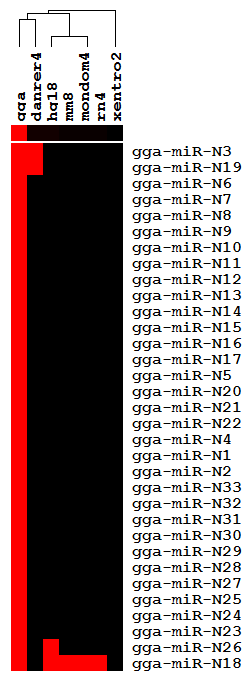

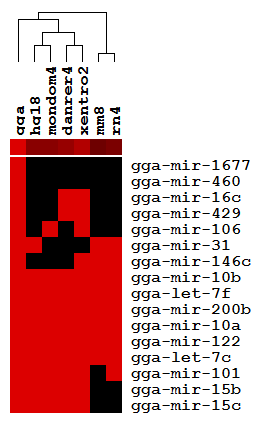


Figure S2 The conservation heat-maps for the known and novel chicken miRNAs. In these two heat-maps, read color means present in the corresponding species and black means absent in the corresponding species. In the heat-map, the dark color represents 0, meaning no homologs were found in the corresponding species. The red color represents 1, which indicates the presence of homologs in the corresponding species.
