# Supplementary figures and images for: A systematic analysis of the skeletal muscle miRNA transcriptome of chicken varieties with divergent skeletal muscle growth identifies novel miRNAs and differentially expressed miRNAs
Source: BMC Genomics. 2011 Apr 13;12:186. doi: 10.1186/1471-2164-12-186 (PMC3107184; doi:10.1186/1471-2164-12-186)

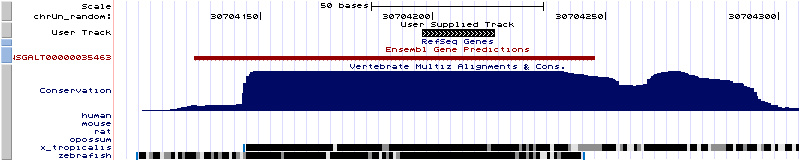


Figure S3. UCSC genome browser tracks showing the conservation of gga-miR-N3.

Supplement: Additional file 7 — Figure S3: UCSC genome browser tracks showing the conservation of gga-miR-N3. [file 1471-2164-12-186-S7.DOC]
