# Supplementary material for: A systematic analysis of the skeletal muscle miRNA transcriptome of chicken varieties with divergent skeletal muscle growth identifies novel miRNAs and differentially expressed miRNAs
Source: BMC Genomics. 2011 Apr 13;12:186. doi: 10.1186/1471-2164-12-186 (PMC3107184; doi:10.1186/1471-2164-12-186)
Supplement: Additional file 14 — Figure S4: Size distribution of reads whose 5' 19 nt were exactly matched to the 5' 19 nt of known miRNAs. [file 1471-2164-12-186-S14.DOC]

Figure. S4 Size distribution of reads whose 5’ 19 nt were exactly matched to the 5’ 19 nt of known miRNAs.
